# Failure characteristics of rocks with non-persistent joints under local load

**DOI:** 10.1371/journal.pone.0291467

**Published:** 2023-09-28

**Authors:** Hui Cheng, Hongbao Zhao

**Affiliations:** School of Energy and Mining Engineering, China University of Mining and Technology (Beijing), Beijing, China; Xi’an University of Science and Technology, CHINA

## Abstract

Jointed rocks under local load are ubiquitous in civil engineering. The instability and failure of jointed rocks are fatal to engineering safety. This paper numerically investigated the effects of loading area and joint angle on the strength dividing points, energy evolution, and crack distribution characteristics of non-persistent jointed rocks. The results demonstrated that the closer the absolute value of joint angle to 45° and the smaller the loading area, the lower the strength dividing points of rocks. The curves of rock joint angle versus total energy at peak and of elastic energy versus amplitude of post-peak abrupt energy change render a W-shape distribution. Meanwhile, compared with joint angle, loading area has more influence on rock energy input. The larger the loading area, the higher the crack fractal dimension, the crack entropy, and the penetration rate. Tensile cracks outnumber shear cracks when jointed rocks are damaged, and shear cracks increases significantly at the post-peak stage.

## Introduction

**Fig 1:**
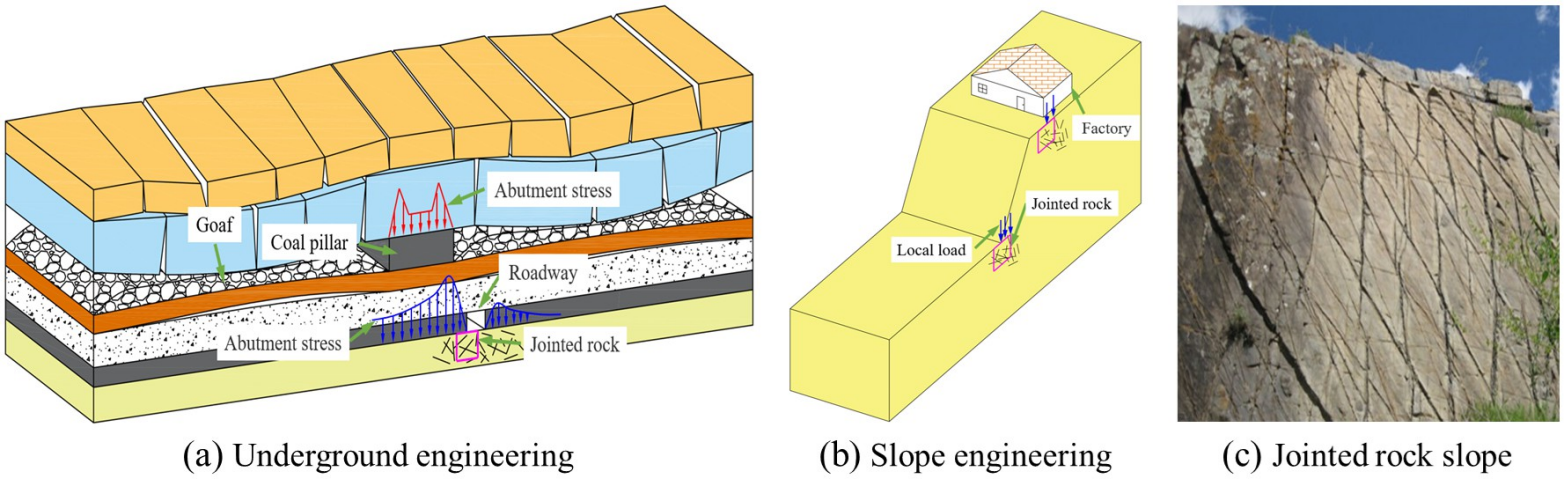
Engineering cases of jointed rocks under local load [1, 2]. (a) Underground engineering, (b) Slope engineering, and (c) Jointed rock slope.

Natural rocks are often prone to damage due to the presence of joints, and the jointed rocks subjected to local load are common in nature (Fig 1). The stability of slopes or masses in an engineering project is related to mechanical behavior of jointed rock under local load. A better understanding of the mechanical behavior and failure characteristics of jointed rocks is required to avoid posing safety risks.

Numerous experimental and numerical investigations have been conducted on the mechanical properties and failure characteristics of jointed rocks. Under uniaxial compression, numerous literatures suggest that the failure characteristics of single jointed rocks are closely related to the mechanical parameters and joint angles of the joint surface [3]. Accordingly, the damage evolution and crack distribution of single jointed rocks were determined and the failure mode summarized. The single jointed rock is classified into tensile failure through joint surface, shear failure along joint surface, tensile failure along joint surface, and complete material failure. The jointed rock damage occurs first at the joints and then throughout the rock [1–7]. The number of joints also has a significant effect on rock failure and mechanical properties, and the failure characteristics of multi-jointed rocks are more complex than those of single jointed rocks [8, 9]. The effects of joint angle, joint parameters, joint spacing, and joint density on the mechanical behavior and failure characteristics of multi-jointed rocks have been investigated by laboratory tests and numerical simulations in numerous literatures [10–13]. It is found that the failure of multi-jointed rocks can be divided into stress-controlled failure, structure-controlled failure, and stress-structure-controlled failure, and the failure type is related to the joint angle [14]. In addition, a series of constitutive models including crack-related parameters was established and verified their proposed models with experimental results to predict the mechanical behavior of jointed rocks [15, 16].

Previous research results are of important guiding significance to the instability and failure prediction of jointed rocks. However, these studies focus on the effects of joint angles, joint density, and joint spacing on the failure characteristics of jointed rocks by mechanical tests and numerical simulation, or on the research and development of constitutive models of jointed rock. In fact, for underground engineering and geotechnical engineering, jointed rocks are used to sustain local load, resulting in serious engineering failures. The effects of loading area on the failure of jointed rocks are rarely reported and further research is needed. Built upon previous research, this paper explored the uniaxial compression of non-persistent jointed rocks under local load, and analyzed the effects of loading area and joint angle on rock stress-strain, strength, energy evolution, and crack distribution. The results provide a theoretical foundation for the failure prediction of non-persistent jointed rocks under local load.

## Simulation test scheme

### Model setting

**Fig 2:**
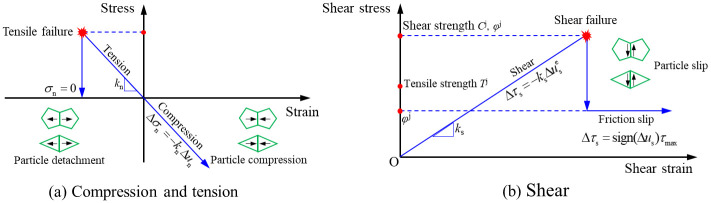
Particle contact constitutive model [21]. (a) Compression and tension and (b) Shear.

**Fig 3:**
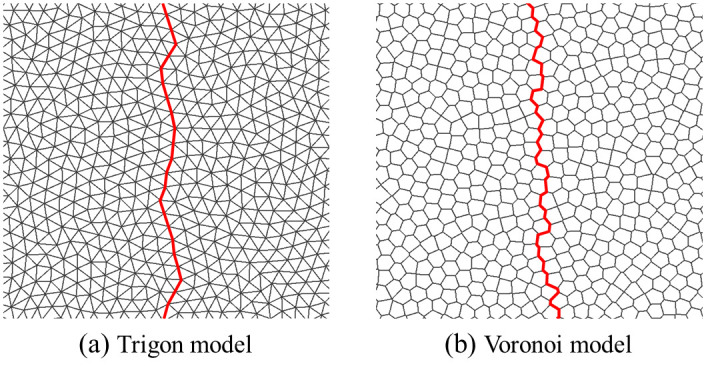
Comparison of crack propagation paths under different particle models. (a) Trigon model and (b) Voronoi model.

Numerically simulating fractures of rock is of great interest in engineering practices and academic studies [17]. UDEC is a discrete element software, which can divide the interior of a rock into polygonal or triangular particles through the Voronoi or Voronoi Trigon command. The contact between particles is called fictitious joint. The deformation of a fictitious joint is characterized by its normal stiffness *k*_n_ and tangential stiffness *k*_s_, whereas the strength is characterized by its friction angle *φ*^j^, cohesion *C*^j^ and tensile strength *T*^j^, with *K* and *G* being the volume modulus and shear modulus of the block. Fig 2 is principle of the particle model. In addition, the shape of particle division has a great influence on crack propagation. Even though the particle shape may not have great influences on mode I cracks, slightly different cracks may cause different mechanical responses, even stress locking, considering mode II and hybrid mode cracks. A lot of studies have alluded to the fact that the Trigon block is less dependent on grids and can obtain a more accurate failure mode of homogeneous rocks [18]. For the crack propagation inside rocks, the path of Voronoi block model is more tortuous, while Trigon block model can better realize the intergranular fracture and transgranular propagation of rocks [19, 20], as shown in Fig 3. Therefore, rocks are divided into Trigon block particles for simulation research.

**Fig 4:**
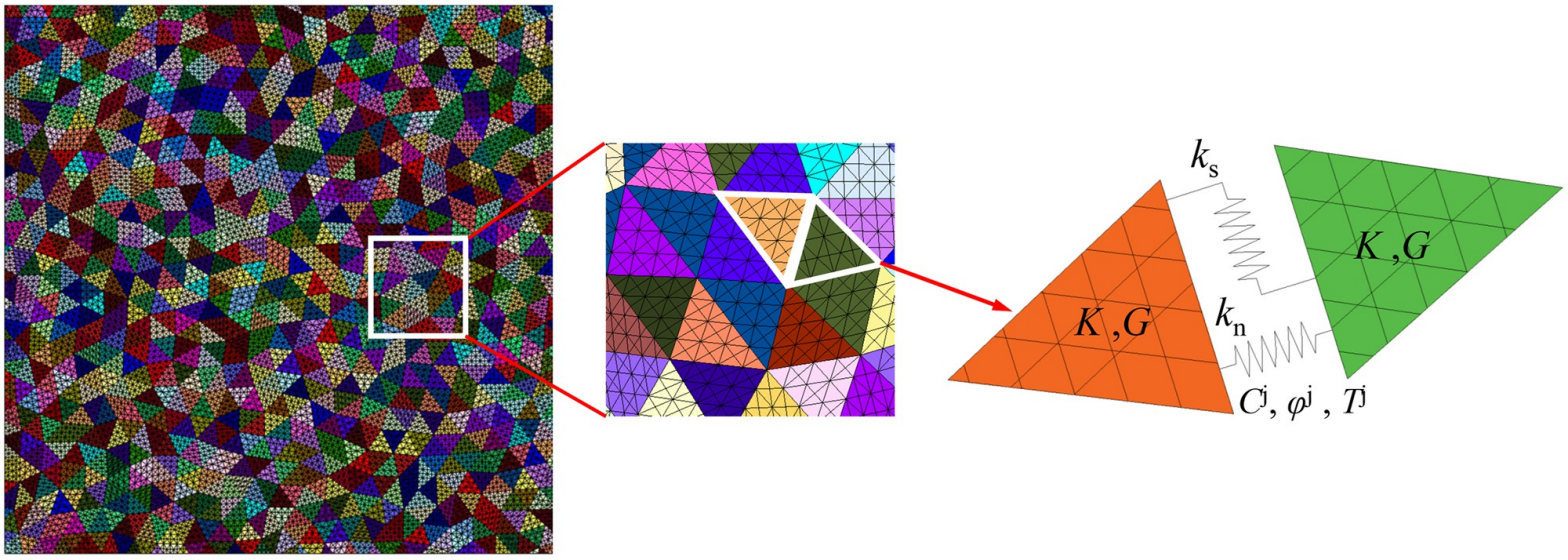
The deformable Trigon block model.

Trigon block particles divided inside the rock are set as deformation bodies to explore the stress and displacement of the rock (Fig 4). For elements in the particles, the material constitutive model is set up as a strain softening model, so as to attenuate rock material parameters. The weakening parameters of the rock after yielding include internal friction angle *φ*, cohesion *C*, dilatancy angle *ψ*, and tensile strength *T*. The fictitious joint is set as the Coulomb friction model to realize the interaction between particles.

### Local load test scheme and parameter calibration of non-persistent jointed rocks

**Fig 5:**
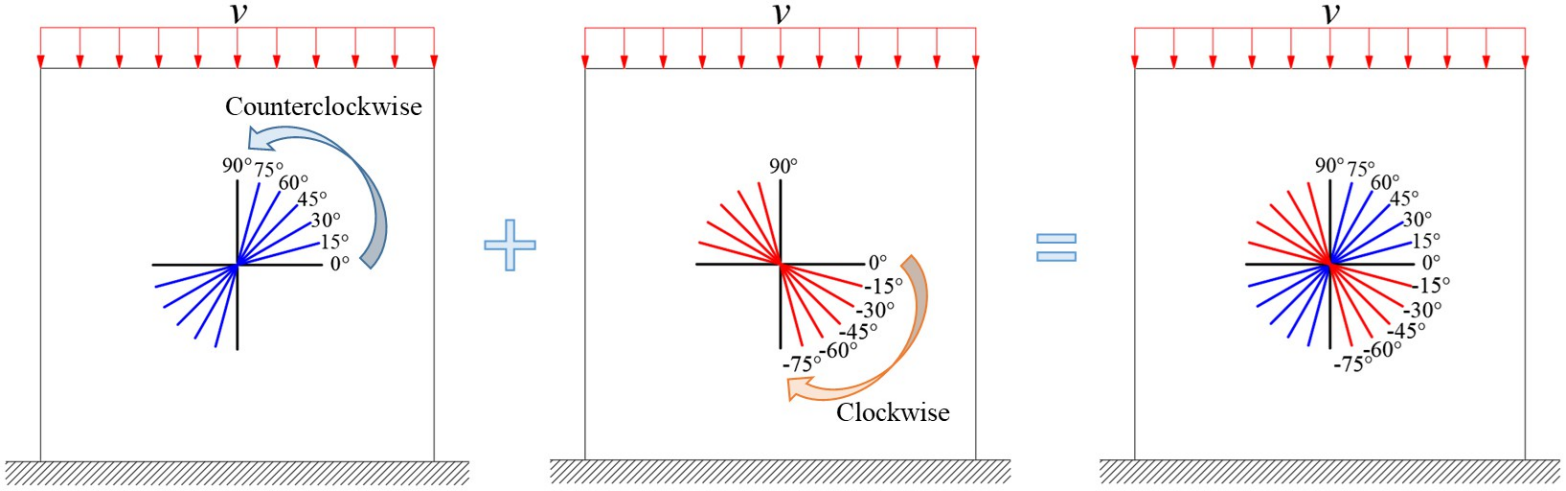
Joint angle schemes.

**Fig 6:**
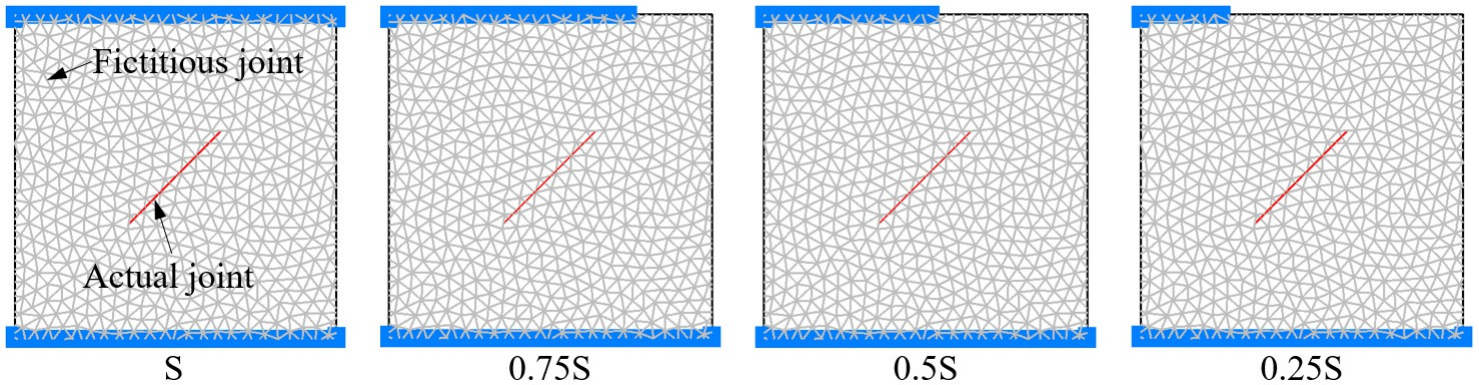
Loading area schemes.

According to the method recommended by ISRM and relevant literature of jointed rock, jointed rock specimens are often set as squares for research [22, 23]. Hence, the rock is designed to be of size 70 mm × 70 mm in UDEC. the joint length is set as 30 mm, distributed in the middle of the model, and the joint angle *α* between the joint and the horizontal direction is set as 0°, 15°, 30°, 45°, 60°, 75°, 90°, -15°, -30°, -45°, -60°, -75° in turn. There are 12 schemes in total, as shown in Fig 5 (Take the positive value for the joint angle *α* if it rotates counterclockwise from the horizontal direction. When joint rotates clockwise, *α* is negative value). Assuming that the end face area of the rock model is S, the local load scheme is divided into four schemes: S, 0.75S, 0.5S, and 0.25S. Uniaxial compression tests were carried out under 4 loading areas for jointed rock with different angle, resulting in a total of 48 simulation schemes. Fixed constraints are set at the bottom of the rocks, the loading speed is set to 0.05 m/s, as shown in Fig 6.

**Fig 7:**
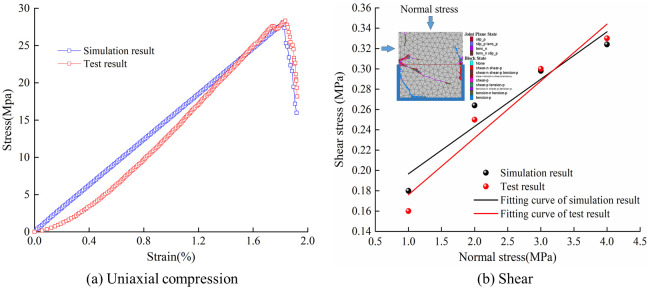
Comparison between laboratory test and numerical simulation. (a) Uniaxial compression and (b) Shear.

Before exploring the mechanical behavior and failure characteristics of jointed rocks, it is necessary to conduct mechanical tests and simulation tests on intact rocks to obtain the model parameters. In UDEC, the microparameters of block and fictitious joint are modified by tring until the stress-strain curve of the rock obtained by numerical simulation is consistent with the test result. The actual joint parameters can be obtained from mechanical shear tests on rocks with single joint by tring until the simulation result match the test result [19]. Taking the rocks of a mine in Shanxi Province as the case study, the uniaxial compression, shear tests, and simulation parameter calibration were performed. The final microparameters are shown in Table 1, and the results of simulation and experiment are basically consistent (Fig 7).

**Table 1:** Mechanical parameters of jointed rock.

Project	Elastic modulus /(GPa)	Poisson’s ratio	Normal stiffness /(GPa·m^-1^)	Tangential stiffness/(GPa·m^-1^)	Internal friction angle/(°)	Cohesion /(MPa)	Tensile strength (MPa)	Dilatancy angle/(°)
block	2.1	0.25	——	——	38	10.5	7	10
Fictitious joint	——	——	2700	2200	35	7.2	4.5	35
Actual joint	——	——	2000	1500	5	0.4	0.2	7

## Strength evolution law of jointed rocks

### Rock strength division

The peak strength *σ*_cf_ of rocks with non-persistent joints can be obtained from the stress-strain curves. The damage stress *σ*_cd_ and crack initiation stress *σ*_ci_ can be determined by the total volume strain method and crack volume strain method, respectively [24–26]. The elastic volume strain of rock specimens under uniaxial compression is given by [26]:

$$\varepsilon_{\text{ve}}=\frac{1-2\nu }{E}\sigma_{\text{z}}$$ (1)

where *ε*_ve_ is the elastic volume strain; *E* is elastic modulus, MPa; *ν* is poisson’s ratio; *σ*_z_ is the axial stress, Mpa.

The volume strain of rock specimen can be determined from the lateral strain and axial strain [27]:

$$\varepsilon_{\text{v}}=\varepsilon_{\text{axial}}+2\varepsilon_{\text{lateral}}$$ (2)

The crack volume strain of the rocks can be obtained by combining Eqs (1) and (2):

$$\varepsilon_{\text{vc}}=\varepsilon_{\text{v}}-\varepsilon_{\text{ve}}$$ (3)

where *ε*_v_ is the rock volume strain; *ε*_axial_ is the axial strain; *ε*_lateral_ is the lateral strain; *ε*_vc_ is the crack volume strain.

**Fig 8:**
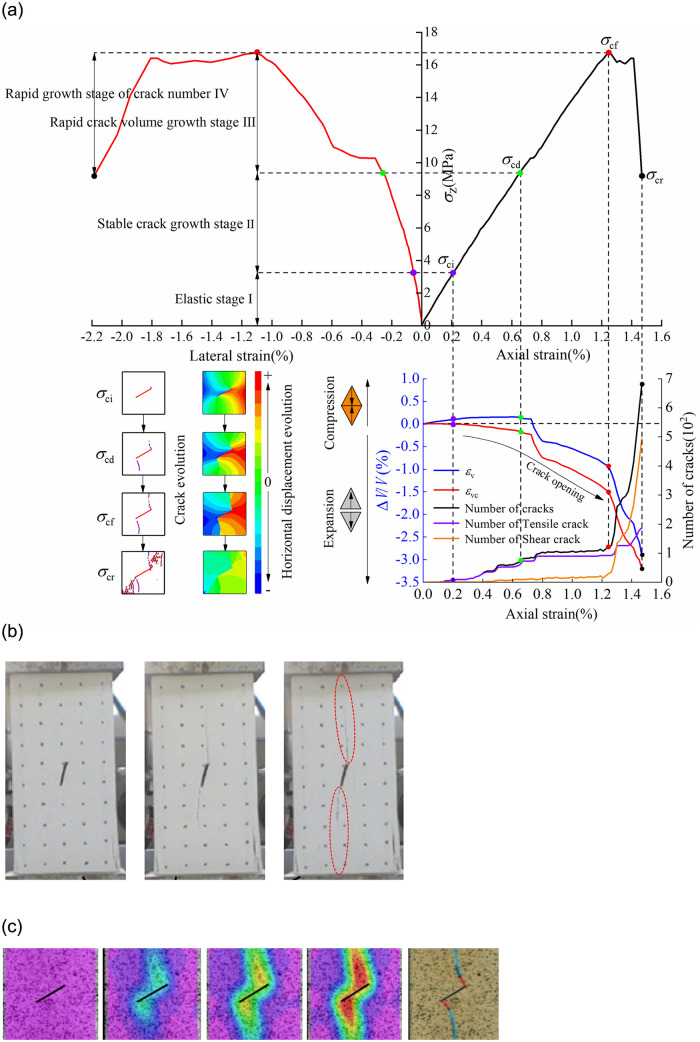
Numerical simulation results and validation. (a) Determination and division basis of strength dividing points of jointed rock with a 30° joint under the full-area load of S, (b) The cracks evolution of jointed rock under uniaxial compression in reference [4], and (c) Strain evolution of 30° jointed rocks under uniaxial compression in reference [30].

*σ*_ci_ can also be obtained from the monitoring results of the number of cracks in the simulation. *σ*_ci_ is consistent, whether obtained by the crack volume strain method or by the crack number monitoring method. *σ*_cd_ is the axial stress corresponding to the reverse bending point of the rock volume strain curve [28]. Due to the similarity in simulation results, taking the rocks with a 30° non-persistent joint under the full-area load of S as examples, the dividing points and division basis of various strengths are shown in Fig 8.

According to the strength dividing points, the stress-strain of the rocks with non-persistent joints can be divided into four segments, each corresponding to a different stage (Fig 8). (1) Stage I: initial crack compaction and elastic stage. Since the initial crack in the rocks occupies no volume in the numerical simulation, the compaction stage is not obvious. As the rock enters the elastic stage, there remains no microcrack in the rock, with *ε*_v_ getting into a state of slow compression. (2) Stage II: slow crack growth stage. Microcracks sprout and expand in the rock, and wing cracks appear at both ends of the joint. Meanwhile, *ε*_vc_ increases slowly. (3) Stage III: rapid crack volume growth stage. The number of cracks in rock increases more rapidly at this stage than at stage II. The wing cracks continue to expand. Both *ε*_vc_ and *ε*_v_ increase rapidly, as manifested in the opening of rock cracks. According to the literatures, no intact rocks undergo this third stage [29], which is a unique feature to jointed rocks. (4) Stage IV: stage of rapid growth of cracks. As *ε*_vc_ and *ε*_v_ increase rapidly, the strength of rocks decreases sharply, the number of cracks, especially of shear cracks, inside the rocks increases sharply, while the number of tensile cracks increases steadily. Fig 8 also shows the failure and displacement characteristics of jointed rocks under full area uniaxial compression. The rock failure mode is consistent with the test results in the literatures [4, 30], which nicely validates the numerical model.

**Fig 9:**
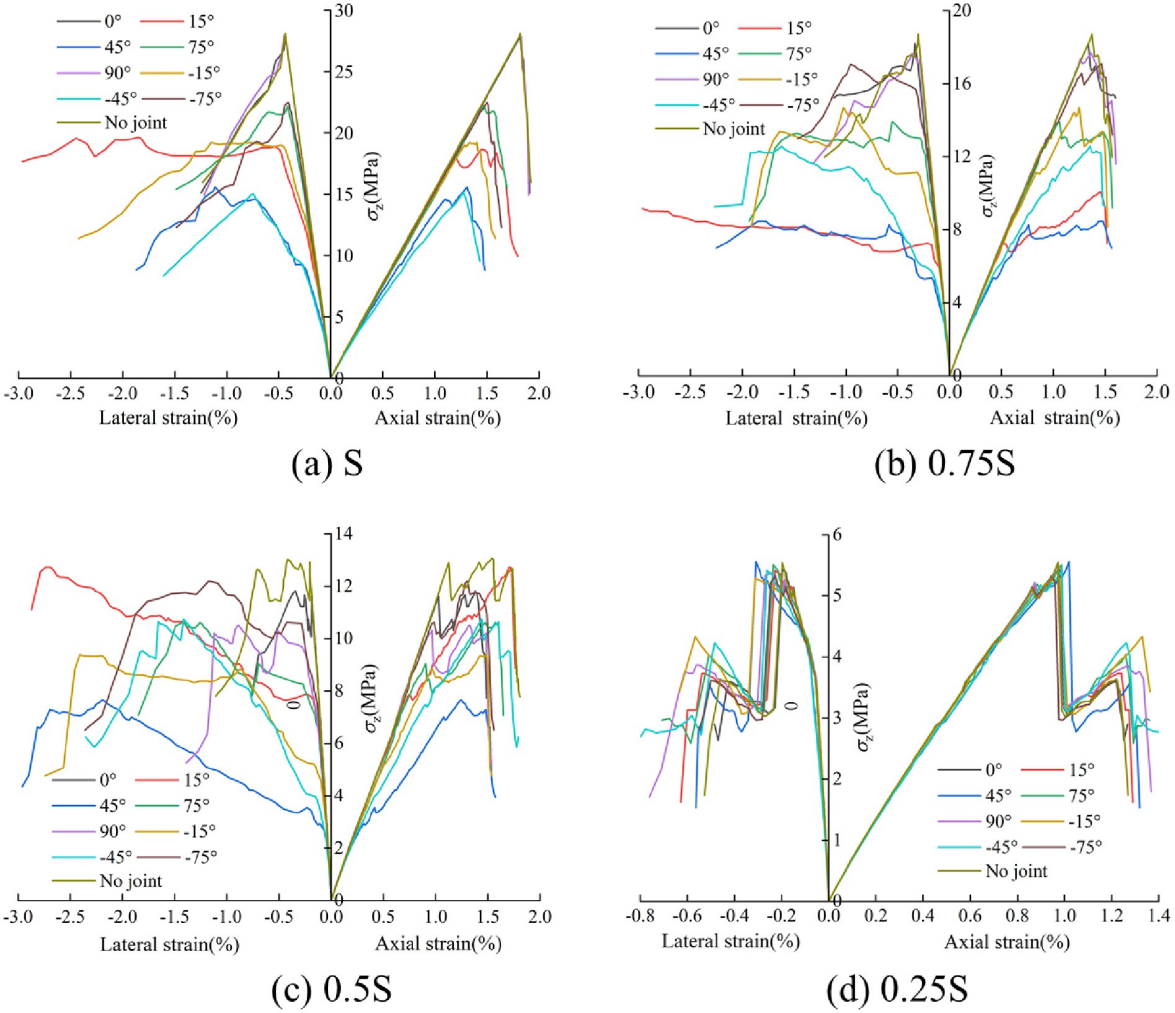
Stress-strain curves of jointed rocks under different loading areas. (a) S, (b), 0.75S, (c) 0.5S, and (d) 0.25S.

The stress-strain curves of jointed rocks under different loading areas were plotted through numerical simulation (Fig 9). As joint angles and loading areas vary, various strength dividing points of rocks undergo significant change, and the crack distribution and horizontal displacement field characteristics are also different.

### Effect of joint dip angle on rock strength

**Fig 10:**
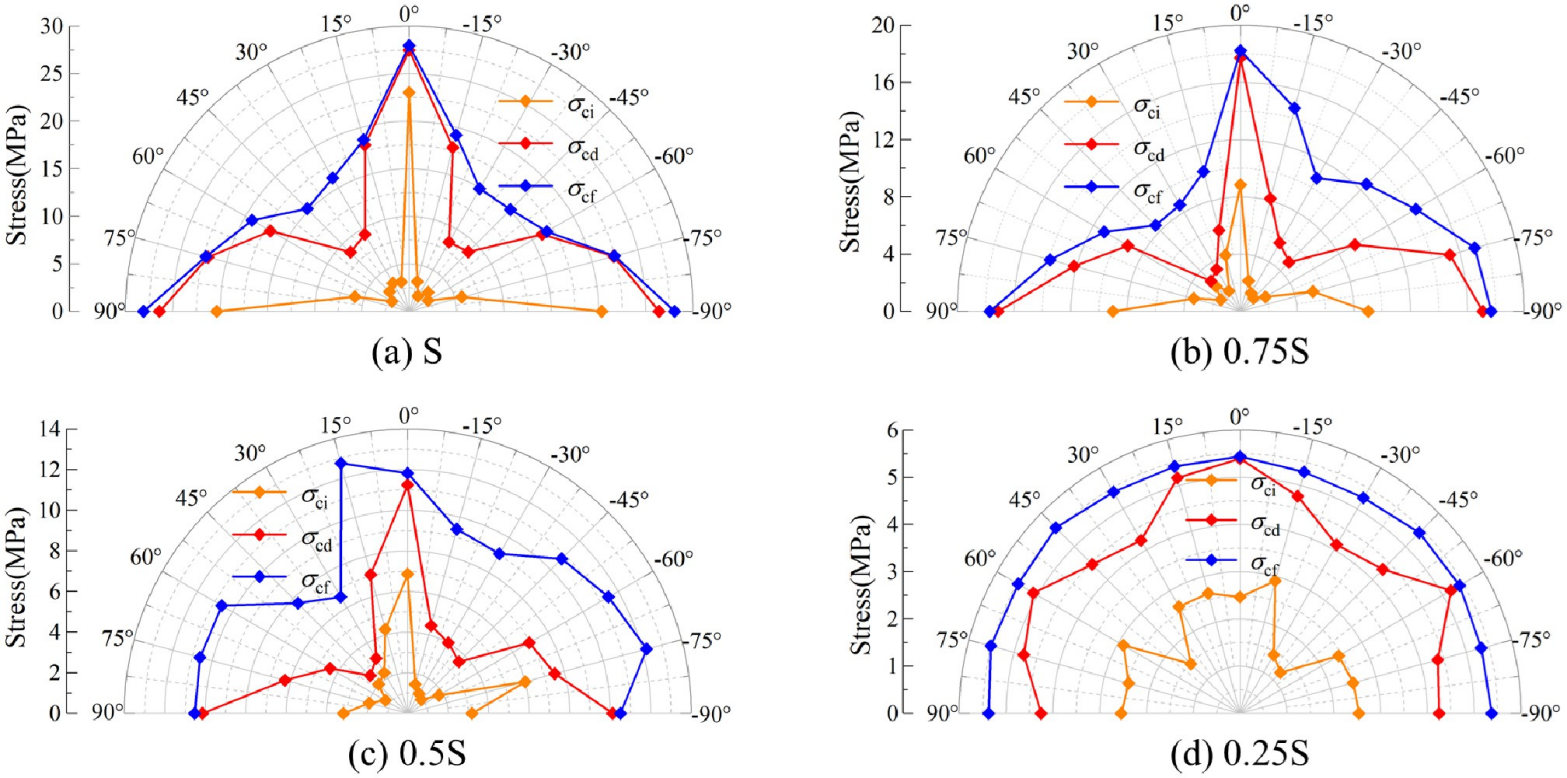
Effect of joint dip angles on strength dividing points of rocks. (a) S, (b), 0.75S, (c) 0.5S, and (d) 0.25S.

Fig 10 shows the effect of joint dip angles on strength dividing points of rocks.

**Fig 11:**
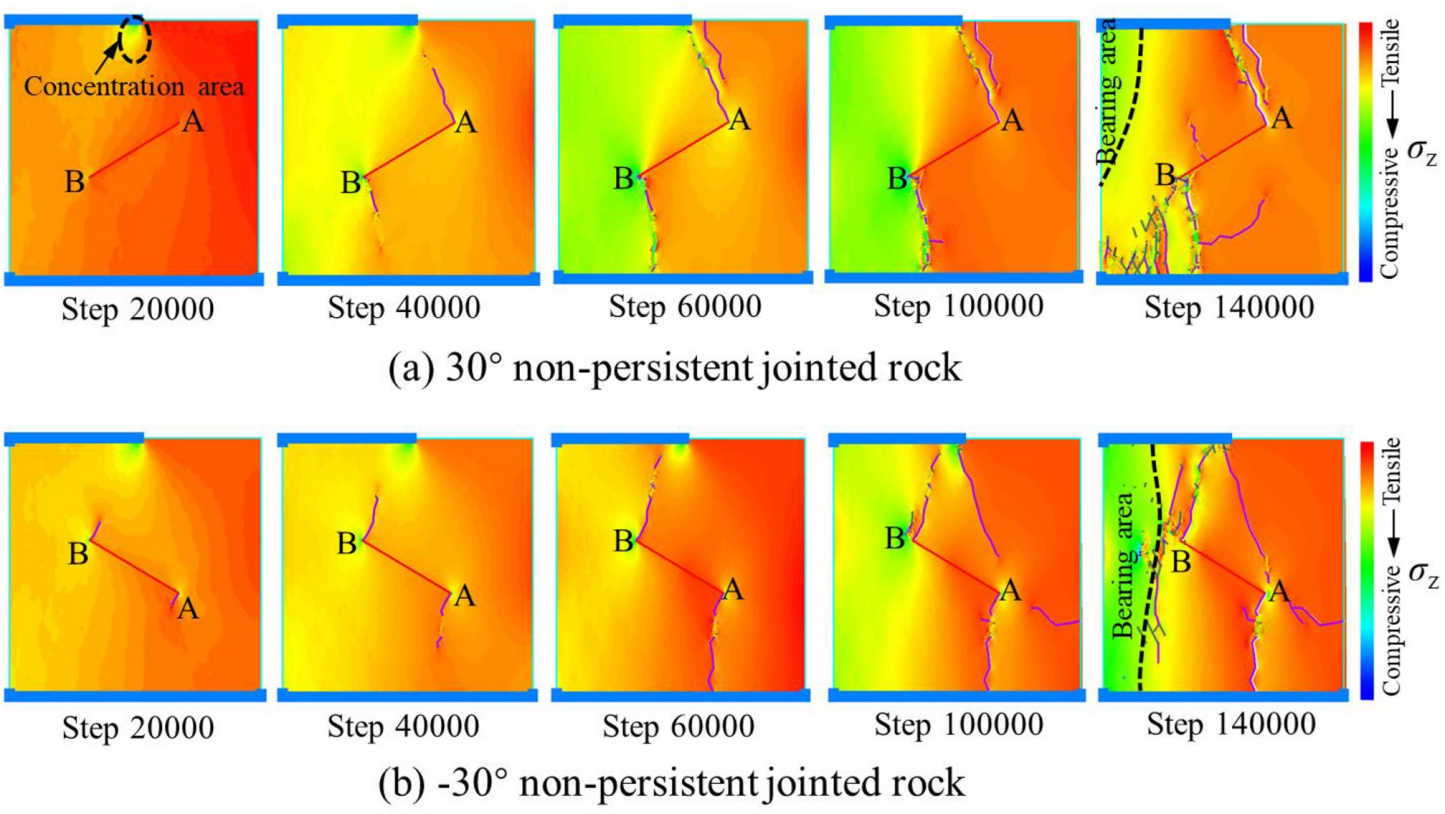
Failure evolution of rocks with same absolute value of joint angle under the half-area load of 0.5S. (a) 30° non-persistent jointed rock and (a) -30° non-persistent jointed rock.

Under the full-area load of S, the strength dividing points reach the minimum at the joint angle of ±45°. The curves of the strength dividing points of rocks are distributed symmetrically about 0°, indicating that under uniaxial compression and at the same absolute value of joint angle, the difference among strength dividing points is nonsignificant. Under the loading areas of 0.75S and 0.5S, each strength stress reaches the minimum for the absolute value of joint angle between 30° and 45°. The strength distribution of rocks under local load appears asymmetric. In general, at the same absolute value of joint angle, the strength dividing points of rocks with a negative joint angle are larger than those with a positive joint angle. For example, under the half-area load of 0.5S, *σ*_cd_ and *σ*_cf_ of one rock with a -30° non-persistent joint angle is greater than their counterparts of another with a 30° non-persistent joint angle (Fig 11).

Under local load, the rock is divided into loading area and non-loading area according to the loading area. The black elliptical dashed line in Fig 11 represents the stress concentration area. A and B represent both ends of the joint. For the 30° non-persistent jointed rock, end B of the joint is located in the loading area, and wing cracks are easy to form at the joint tip. Although end A is located in the non-loading area, wing cracks are also easy to form and expand towards the stress concentration area. For the -30° non-persistent jointed rock, end B of the joint is not only located in the loading area but also subject to the stress concentration area. It is very easy to crack under the combined action. Therefore, *σ*_ci_ of the -30° non-persistent jointed rock is smaller than that of the 30° non-persistent jointed rock. At 40000th step, obvious wing cracks form and expand at the joint, making the rock more vulnerable to damage. While the -30° non-persistent jointed rock has obvious wing cracks expanding only at end B, the wing crack propagation is not obvious at end A. Therefore, *σ*_cd_ of the -30° non-persistent jointed rock is greater than that of the 30° non-persistent jointed rock. At the 140000th step, tensile cracks form at end A of the 30° non-persistent jointed rock, while tensile wing cracks, secondary tensile cracks, and main shear cracks form at end B of joints. The range of rock stress bearing area is greatly narrowed. While tensile wing cracks form at end A of the -30° non-persistent jointed rock, wing cracks and secondary tensile cracks form at end B. Still having a certain range of bearing area, the rock can continue to bear pressure. Therefore, *σ*_cf_ of the -30° non-persistent jointed rock is greater than that of the 30° non-persistent jointed rock.

Under the loading area 0.25S, *σ*_cf_ of rocks is symmetrically distributed about 0°. Moreover, both *σ*_ci_ and *σ*_cd_ are smaller when the joint dip angle is close to ±45° since end B of the joint is in the non-loading area. The wing cracks cause no structural damage to the jointed rocks, but they impose uniaxial compression on the loading area. Therefore, *σ*_cf_ is basically the same.

### Effect of loading area on rock strength

**Fig 12:**
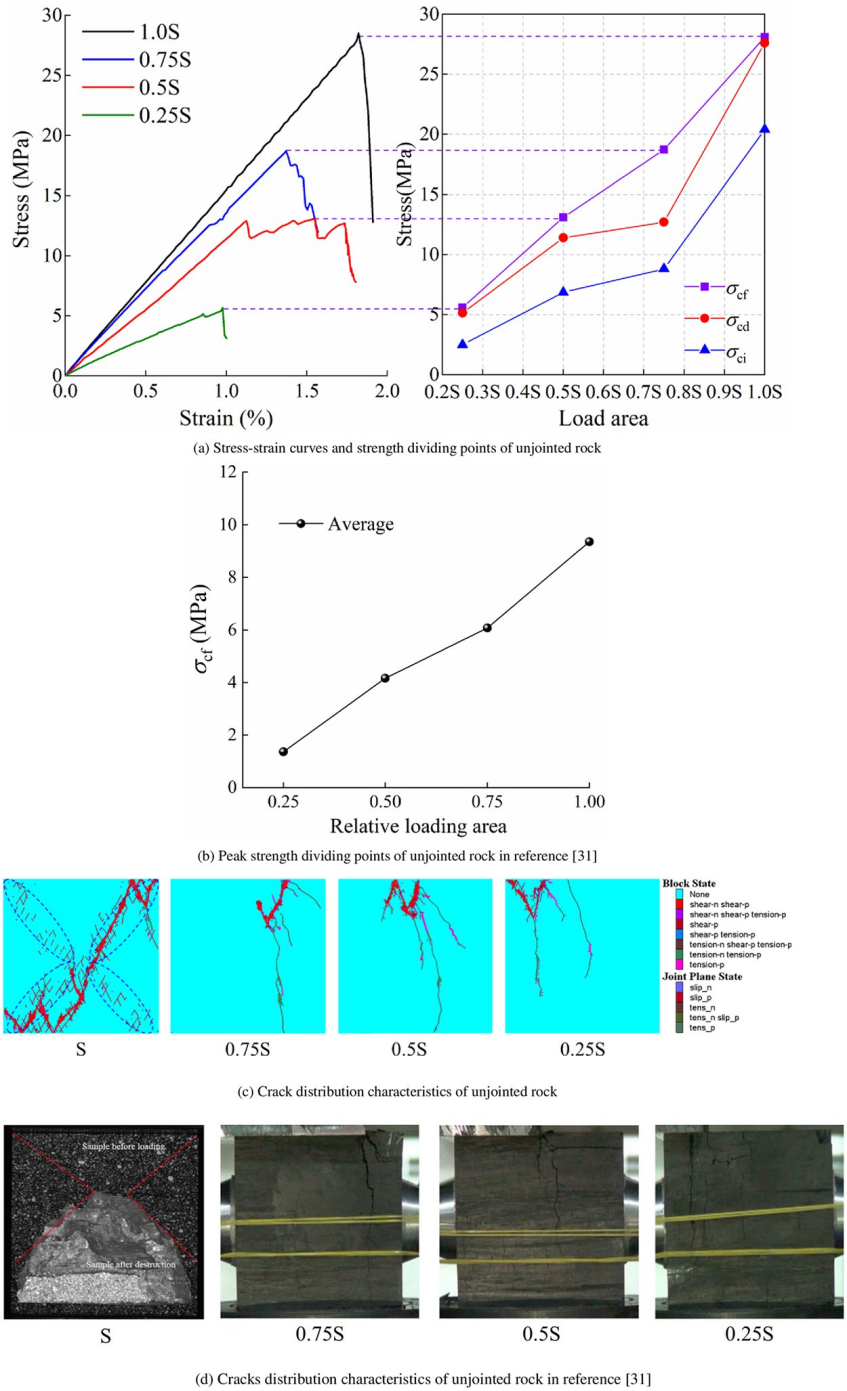
Simulation results and validation of strength dividing points and cracks distribution of unjointed rock. (a) Stress-strain curves and strength dividing points of unjointed rock, (b) Peak strength dividing points of unjointed rock in reference [31], (c) Crack distribution characteristics of unjointed rock, and (d) Cracks distribution characteristics of unjointed rock in reference [31].

**Fig 13:**
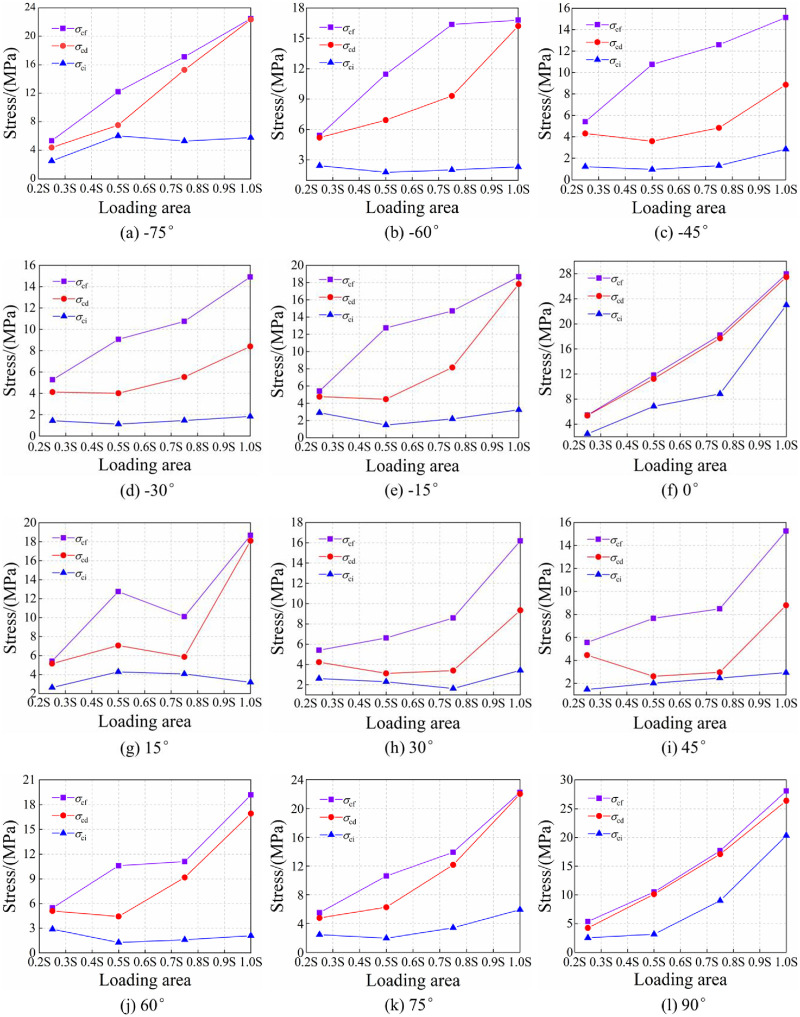
Strength dividing points of jointed rocks with different angles under different loading area. (a) -75°, (b) -60°, (c) -45°, (d) -30°, (e) -15°, (f) 0, (g) 15°, (h) 30°, (i) 45°, (j) 60°, (k) 75°, and (l) 90°.

Fig 12 shows the strength dividing points evolution laws of unjointed rocks under different loading areas. *σ*_ci_, *σ*_cd_, and *σ*_cf_ of unjointed rocks are related with the loading area. The larger the loading area, the higher the strength dividing points of rocks, and the higher dispersion degree of stress strength. The effect of loading area on the mechanical behavior of unjointed rocks is consistent with the results in the literature [31]. This is manifested as the strength of unjointed rocks increases with the increase in loading areas. Meanwhile, the crack distribution characteristics of unjointed rock obtained from simulation and test are consistent, which once again verifies the reliability of the numerical model. The strength dividing points evolution laws of jointed rocks with different joint angles under different loading areas are shown in Fig 13.

As shown in Fig 13, the strength division points of non-persistent jointed rocks at different angles usually monotonically increases with the increase in loading area. When the joint angle is 0° or 90°, *σ*_ci_ of rocks under different loading areas disperses largely. When the joint angle is neither 0° nor 90°, *σ*_ci_ of rocks changes little and disperses to a small degree under different loading areas. When the joint angle is close to ±45°, *σ*_cd_ is minimized at the half-area load of 0.5S. When the joint angle is closer to the 0° or 90°, *σ*_cd_ renders a monotonically increasing tendency with loading area, maximized at the full-area load of S. *σ*_cf_ of rocks and loading area are related generally by monotonically increasing relationship.

## Energy evolution law of jointed rocks

The failure and deformation of the rock mass are mainly driven by energy [32, 33]. In the loading process of rocks, the input energy is transformed mainly into the elastic strain energy and the dissipative energy [33–35]. By the first law of thermodynamics, ignoring the heat exchange between rocks and the outside world, it can be considered that the input energy of jointed rocks is transformed entirely into the elastic strain energy and the dissipative energy:

$$U=U_{\text{d}}+U_{\text{e}}$$ (4)

where *U* is the total strain energy, kJ/m^3^; *U*_d_ is the dissipative energy, kJ/m^3^; *U*_e_ is the elastic strain energy, kJ/m^3^.

Under uniaxial compression, *U* of rocks is expressed as [36–38]:

$$U=\int \sigma_{\text{z}}\text{d}\varepsilon_{\text{z}}$$ (5)

where d*ε*_z_ is axial strain increment.

By Hooke’s law, *U*_e_ of jointed rocks can be derived:

$$U_{\text{e}}=\frac{1}{2}\sigma_{\text{z}}\varepsilon_{\text{z}}^{\text{e}}\approx \frac{\sigma_{\text{z}}^{2}}{2E_{0}}$$ (6)

where *E*_0_ is the initial elastic modulus of rock, MPa.

**Fig 14:**
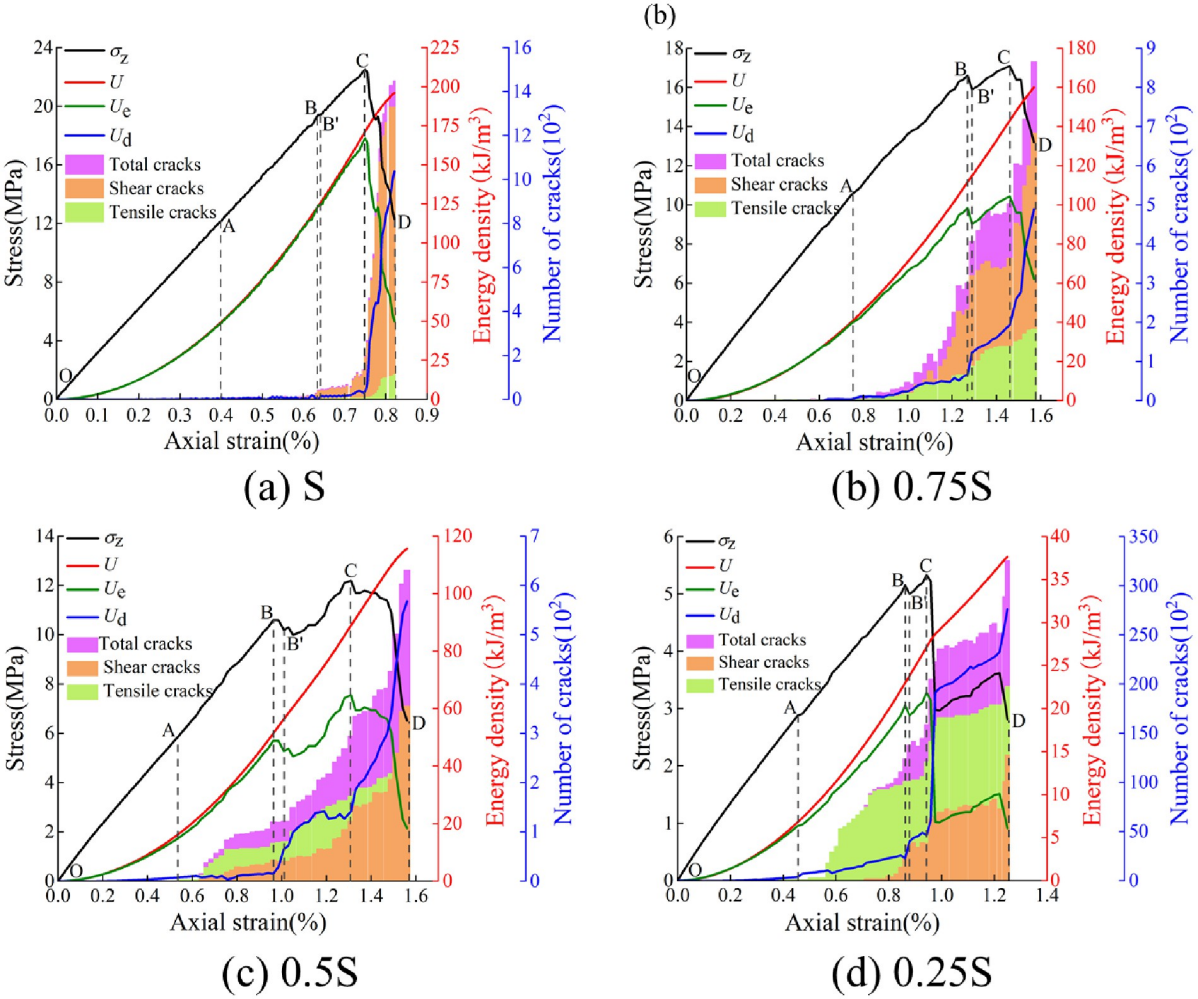
Energy and crack evolution law of -75° non-persistent jointed rock under different loading areas. (a) S, (b), 0.75S, (c) 0.5S, and (d) 0.25S.

Combining Eqs (4)–(6), the energy evolution law of jointed rocks can be determined. Due to the similarity in energy and crack evolution of jointed rock, taking the -75° non-persistent jointed rock as example, as shown in Fig 14.

The energy evolution of jointed rocks under local load falls into five stages. (1) Elastic energy storage stage (OA). The rocks are in an elastic state, where *U* is transformed into *U*_e_ and stored in rocks, while *U*_d_ is basically zero. The slope of the total energy curve increases gradually with the increase of strain, and the energy accumulation capacity of the rocks is enhanced. (2) Pre-peak nonlinear energy storage stage (AB). *U* is transformed mainly into *U*_e_, where the rocks suffer a damage due to the crack initiation and expansion in them. The number of tensile cracks in jointed rocks increases sharply, while the shear cracks grows slowly. At this stage, *U*_d_ increases slowly, and *U*_d_ << *U*_e_. (3) Pre-peak energy jump dissipation stage (BB’). At this stage, *U* increases linearly, while *U*_e_ and *U*_d_ drop or increase by leaps and bounds, as embodied in the sudden drop of *U*_e_ and sudden increase of *U*_d_. Notwithstanding the increasing numbers of shear cracks and tensile cracks in the rocks, *U*_e_ remains greater than *U*_d_. (4) Pre-peak acceleration dissipation stage (B’C). At this stage, *U* increases linearly, and *U*_e_ remains on increase. *U*_d_ and dissipation rate of the rocks both increase significantly. Crack initiation is further enhanced, expanding and penetrating through the rocks, so that the bearing capacity of rocks reach the limit. (5) Post-peak failure energy dissipation stage (CD). At this stage, *U* is supplied at a low rate, and *U*_e_ is on the sharp decrease while *U*_d_ is on the sharp increase, with the latter outweighing the former. The cracks continue expanding and penetrating at a higher rate, so that the shear cracks inside the rocks increases rapidly and that the strength of the rocks decreases sharply. For the jointed rocks under local load, before the stage CD, the interior of the rocks is predominated by tensile cracks, which significantly outnumber shear cracks. In the stage CD, under the load areas of S, 0.75S, and 0.5S, the number of shear cracks is significantly larger than that of tensile cracks. Under the quarter-area load of 0.25S, the interior of the rocks remains predominated by tensile cracks, which significantly outnumber shear cracks.

**Fig 15:**
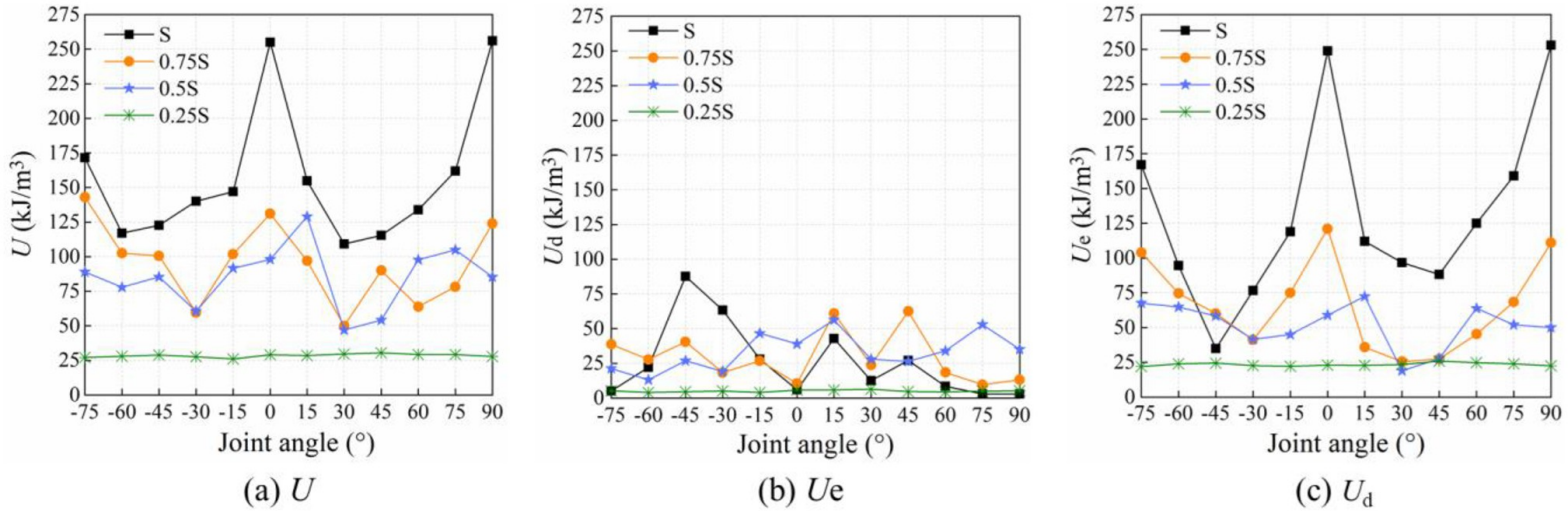
Relation curves between joint angle and *U*, *U*_e_ and *U*_d_ at peak strength of rocks under local load. (a) *U*, (b) *U*_e_, and (c) *U*_d_.

Energy drive is responsible for the damage and failure of rocks. Fig 15 shows the distribution of *U*, *U*_e_ and *U*_d_ at peak strength of rocks with different joint angles under local load. *U* and *U*_e_ at peak strength are both indicative of the difficulty of damage and failure of jointed rocks driven by energy. The greater the *U* and *U*_e_ at peak strength, the more energy it takes for damage and failure to occur inside the rocks. Under the full-area load of S, the relationship between joint angle and *U* and *U*_e_ at peak strength show a W-shape distribution. Under the loading areas of 0.75S and 0.5S, the above-mentioned relationship curves also render a W-shape distribution, except that the energy difference between rocks with different joint angles decrease, indicating that the effect of loading area on energy input is dominant while the effect of joint angle of rocks is weakened. Under the loading area of 0.25S, the rocks are hardly subject to joint angle. The energy absorbed or dissipated is basically the same of the rocks with different joint angles.

**Fig 16:**
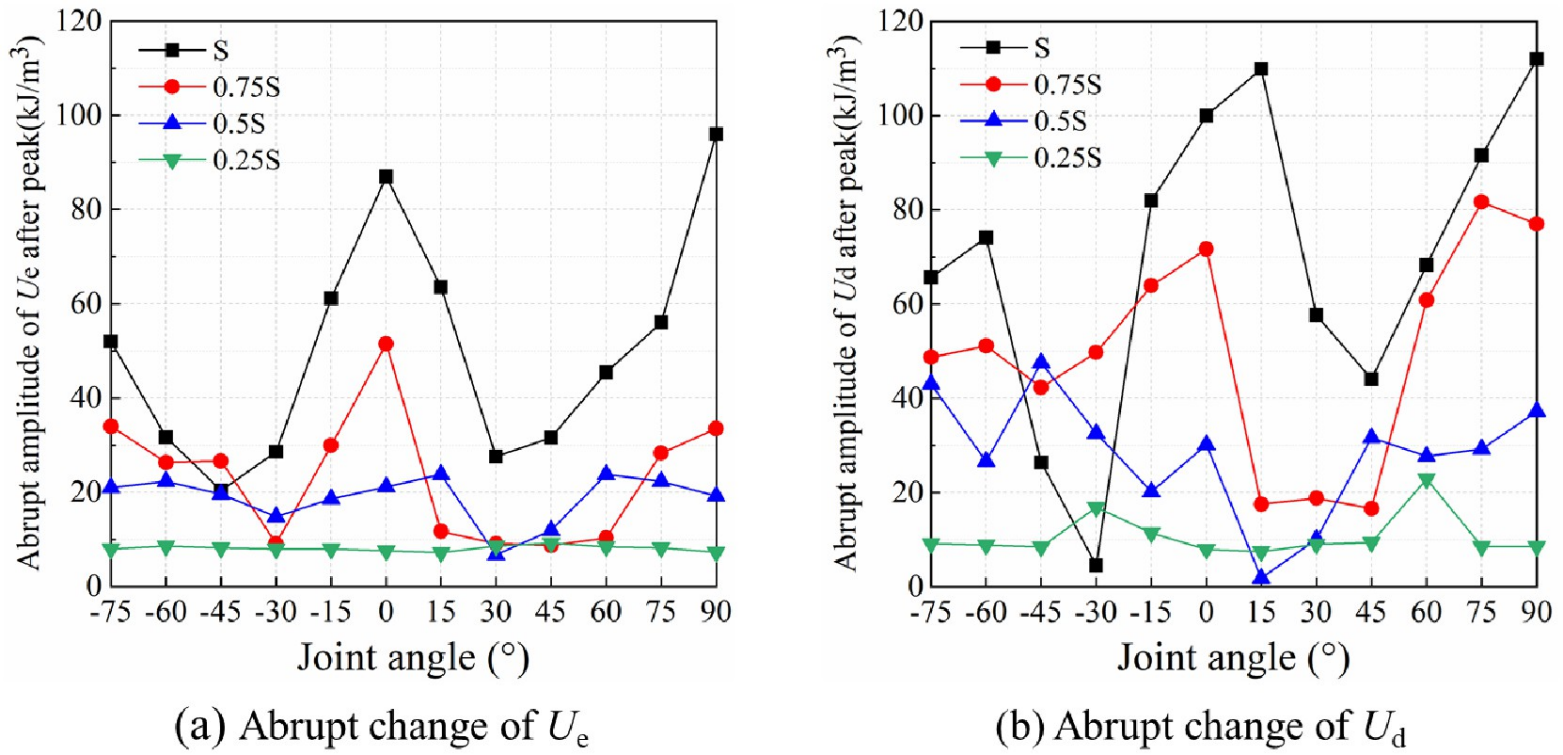
Relationship curves between rock joint angle and amplitude of post-peak abrupt energy change under local load. (a) Abrupt change of *U*_e_ and (b) Abrupt change of *U*_d_.

Fig 16 shows the abrupt changes of *U*_e_ and *U*_d_ of rocks in the post-peak stage under local load. The post-peak characteristic points are 0.8σ_cf_ stress points. The energy difference between the peak point and the post-peak characteristic point is the amplitude of the abrupt energy change. The greater the amplitude of the abrupt post-peak change of *U*_e_ and *U*_d_, the faster the release of energy, and the larger the degree of rock cracking and collapse [39, 40]. Under the full-area load of S, the relation curve between amplitude of the abrupt post-peak energy change of rocks and joint angle presents a W-shape distribution. As the joint angle of rocks gets close to 0° or 90°, the degree of rock cracking and collapse is enlarged. Under the three-quarter-area load of 0.75S, the relation curve between amplitude of the abrupt change and joint angle of rocks still shows a W-shape distribution, except that the difference in the amplitude of the abrupt change between rocks with different joint angles decreases. Under the half-area load of 0.5S, the amplitude of the abrupt energy change of rocks with different angle joints is different, but the difference shows a decreasing tendency. Under the quarter-area load of 0.25S, the amplitude of the abrupt energy change of rocks varies little with joint angle, staying basically the same at different joint angles.

## Crack evolution law of jointed rocks

### Evolution and distribution of cracks in jointed rocks

**Fig 17:**
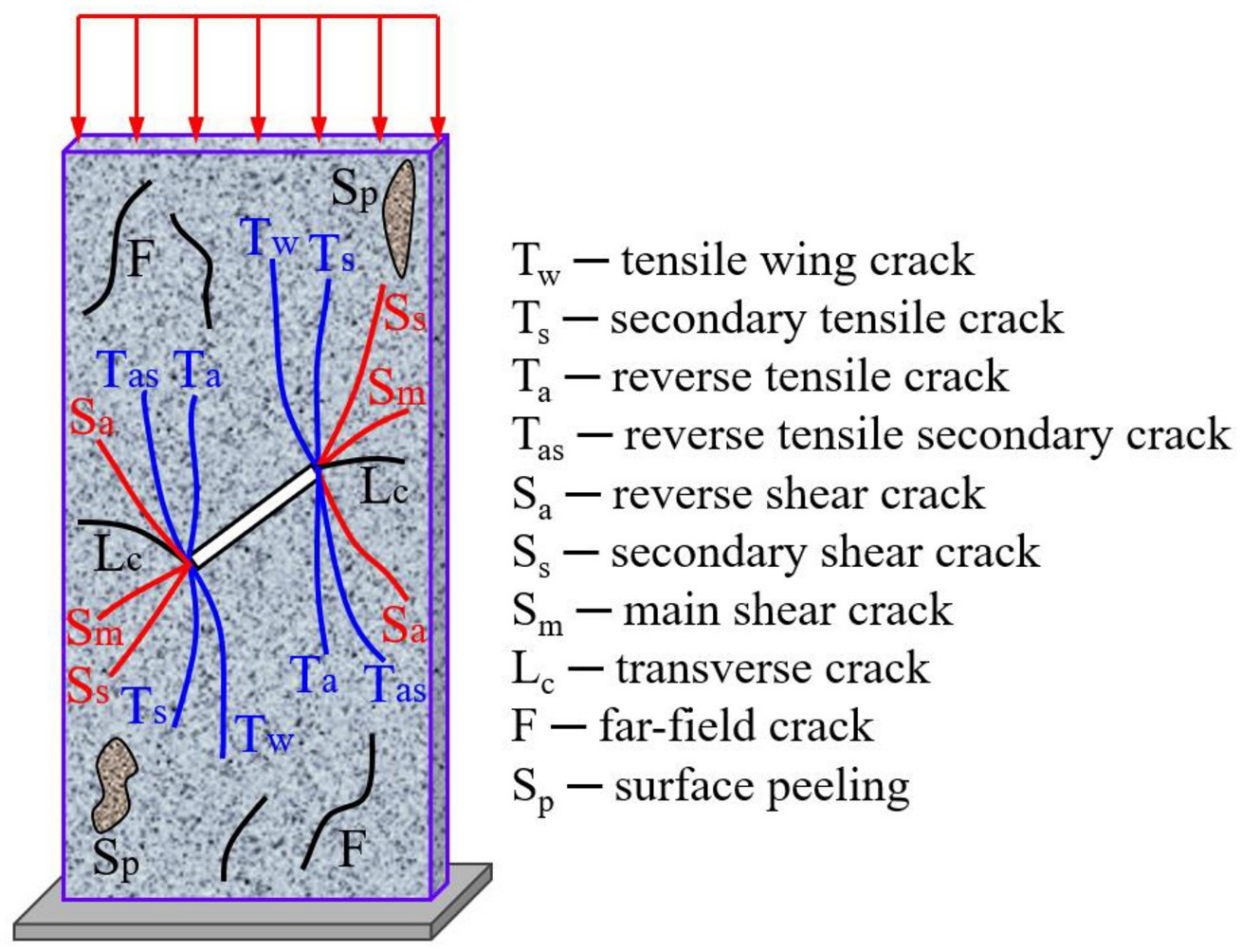
Crack distribution of non-persistent jointed rock [41].

According to the existing research literatures, Fig 17 shows the crack distribution law of non-persistent jointed rocks under uniaxial compression, The crack distribution of jointed rocks under local load is corresponding to Fig 17.

**Fig 18:**
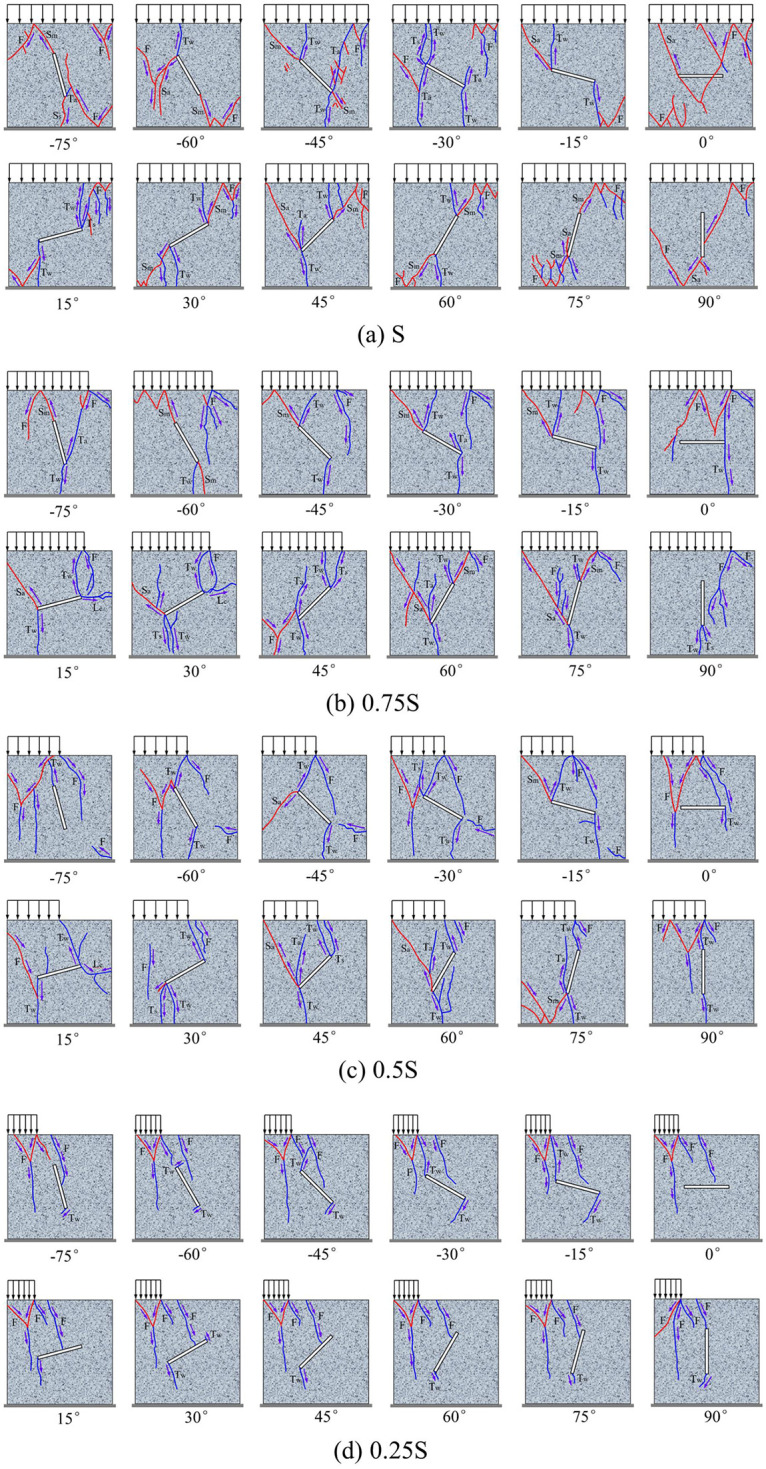
Crack distribution law of jointed rocks under different loading areas. (a) S, (b), 0.75S, (c) 0.5S, and (d) 0.25S.

The post-peak failure crack distribution of jointed rocks under different loading areas are shown in Fig 18. The blue line represents tensile cracks, the red line represents shear cracks, and the arrow indicates the direction of crack propagation.

Under the loading areas of S, 0.75S, and 0.5S, when the absolute value of joint angle |*α*| = 45°, tensile cracks and shear cracks form at both ends of the joint, and crack propagation and penetration lead eventually to the formation of tensile-shear composite failure of jointed rocks. When |*α*| < 45°, tensile cracks form mainly at both ends of the joint, and shear cracks, except for far-field ones, are less likely to form in the rocks. When |*α*| > 45° and *α* ≠ 90°, tensile cracks and shear cracks form at both ends of the rock joint. Macrocracks form as shear cracks initiate and expand at the joint tip, and the jointed rocks mostly suffer a tensile-shear failure. Therefore, as *α* approaches 0°, the macro failure type of rocks joint tip transforms from tensile-shear failure to tensile failure. When *α* = 0°, the existence of joints has deteriorative but limited effect on the rocks. The macro failure type of rocks is immune to joints, but it is mainly subject to the loading area. Under the local loading areas of 0.75S, 0.5S, and 0.25S, the rocks form a stress concentration at the critical point of loading, so that cracks initiate and expand downward to form far-field tensile cracks. When the joint tip is close enough to the critical point of loading, the crack initiation at the joint tip is induced to expand toward that point. In the rock loading area, far-field shear cracks often form in this area, expanding from top to bottom, and the crack type varies from shear to tension, finally forming a Y-shape crack. For example, under the loading area 0.25S, the joint is completely inside the non-loading area. The Y-shape cracks form in the loading area and the far-field tensile cracks form at the loading critical point, causing the macro failure of rocks.

**Fig 19:**
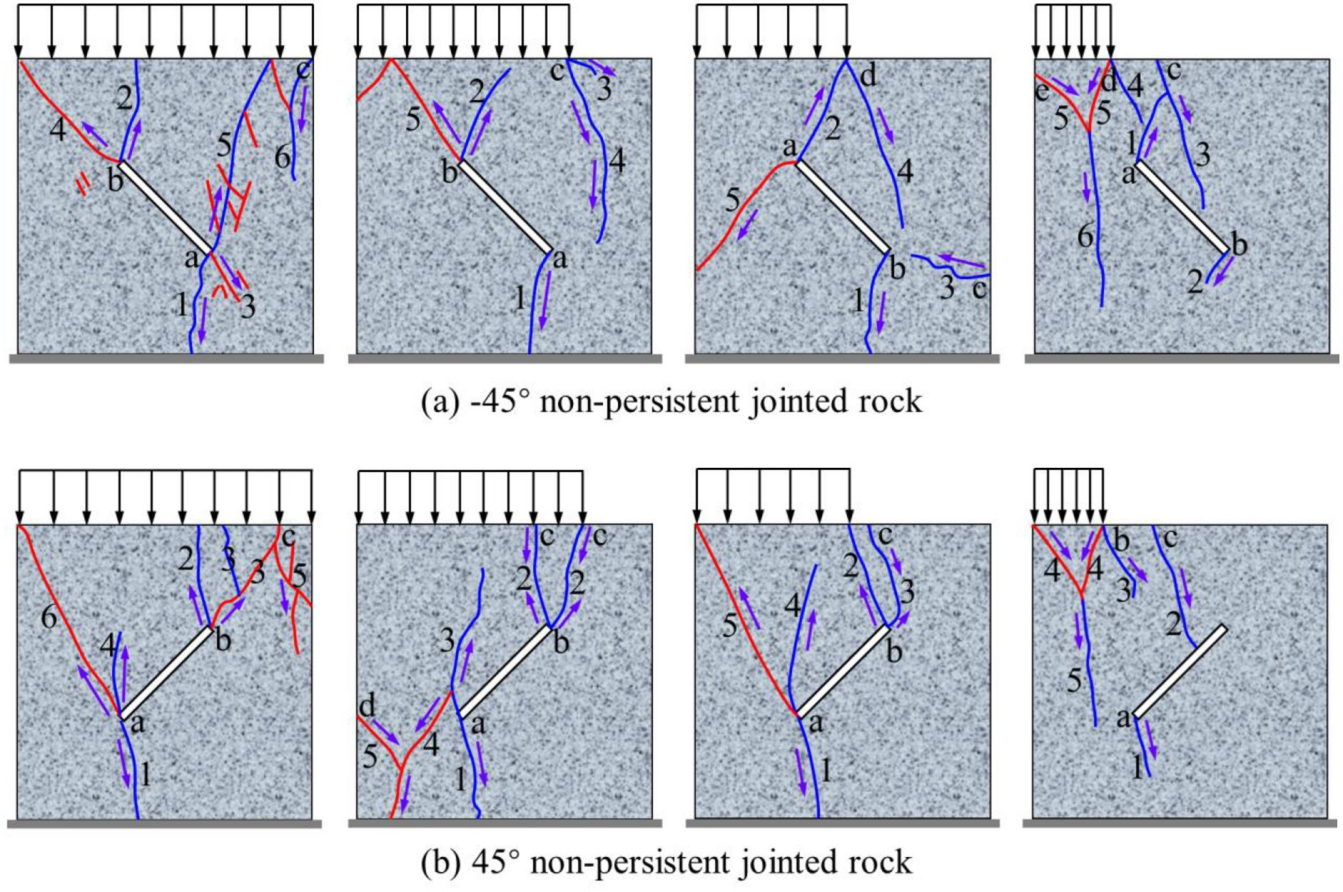
Failure process of non-persistent jointed rock. (a) -45° non-persistent jointed rock and (a) 45° non-persistent jointed rock.

Taking -45° and 45° non-persistent jointed rocks as examples, Fig 19 shows their failure process, in which the sequence of letters a, b, c, d… represents the cracks initiation sequence of rock, and the arabic numerals 1, 2, 3, 4… represent the formation sequence of cracks. It can be seen from the figures that for non-penetrating jointed rocks, one or both ends of the rock joint crack first and form tension wing cracks under any loading area. Then, under the full-area load of S, some shear cracks continue to form at ends of the joint to penetrate the rock, and finally form the tension-shear composite cracks in the far-field. Under the loading area of 0.75S, 0.5S, 0.25S, after the rock forms tensile wing cracks at the joint, the crack initiation point forms at the loading critical point, and the tensile cracks form from top to bottom. Finally, under the loading area of 0.75S and 0.5S, the far-field crack or the shear crack at ends of the joint form in the rocks, while the rock under the loading area of 0.25S form a Y-shaped crack in the loading area.

To sum up, the crack propagation and distribution law of non-persistent jointed rocks under local load are subject to joint occurrence and loading area. The joint angle affects the crack type and distribution characteristics at the joint tip, whereas the loading area changes the direction of crack propagation, cause stress concentration, form tensile cracks, and aggravate rock failure.

### Crack fractal dimension, fracture entropy, and penetration rate of jointed rocks

The fractal dimension, fracture entropy, and crack penetration rate reflect the difference of post-peak failure degree of jointed rocks under local load, among which the fractal dimension reflects the complexity of crack propagation and evolution on rock surface [42]. At present, the box counting is widely used for fractal dimension measurement owing to the simplicity of its principle. This method aims to determine the number of square grids of different sizes required to completely cover the object to be measured through these grids [43, 44]:

lgN(δ)=lga−Dlgδ (7)

where *N* is the number of grids; *a* is a positive constant; *D* is the fractal dimension; *δ* is the grid size, mm.

The information entropy reflects the value of information and the information content in each code sent from the information source, generally defined as [45]:

$$H=-\sum_{i=1}^{n}P_{i}\;\text{ln}\;P_{i}$$ (8)

where *H* is probability entropy; *P*_*i*_ is the probability of a signal, and *n* is the type of signal.

By the principle of information entropy, the fracture entropy is defined by the below formula to reflect the randomness and degree of disorder of crack evolution in jointed rocks [46].

$$K_{f}=\frac{-\sum_{i=1}^{n}\frac{p_{i}}{\sum_{i=1}^{m}p_{i}}\;\text{ln}(\frac{p_{i}}{\sum_{i=1}^{m}p_{i}})}{\text{ln}(n)}$$ (9)

where *K*_*f*_ is the fracture entropy. The greater its value, the more disorderly and more random the distribution of rock fractures; *p*_*i*_ is the probability of cracks forming in a region of rocks; *m* is the total area of the region; *n* is the number of regions divided.

The crack penetration rate reflects the penetration degree and failure degree of rock cracks, and it is defined as:

$$R_{f}=\frac{S_{f}}{S_{a}}$$ (10)

where *R*_*f*_ is the fracture penetration rate; *S*_*f*_ is the crack area, mm^2^; *S*_a_ is the total area of rock surface, mm^2^.

**Fig 20:**
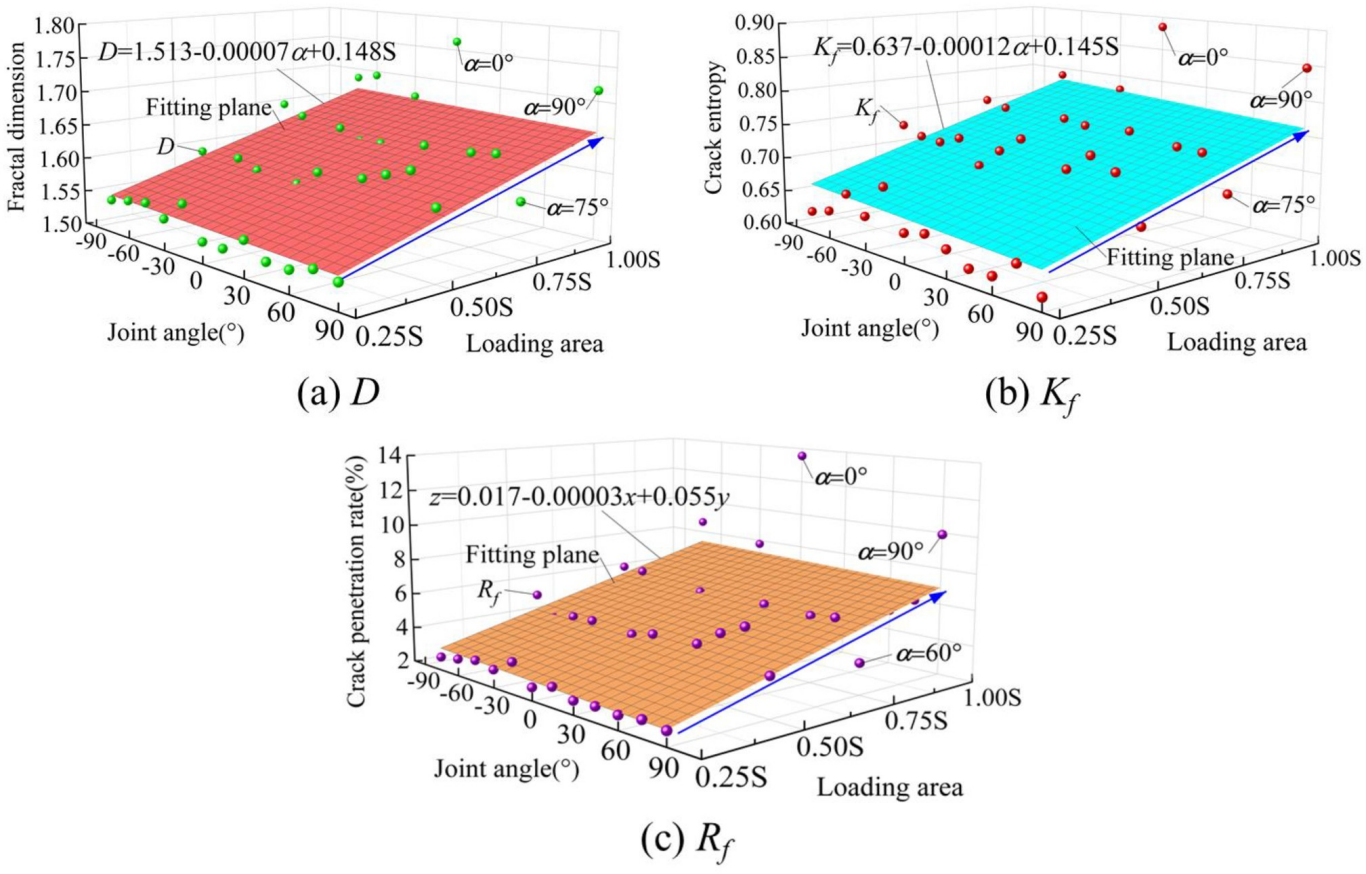
Distribution law of *D*, *K*_*f*_, and *R*_*f*_ of post-peak cracks in jointed rocks under local load. (a) *D*, (b) *K*_*f*_, and (c) *R*_*f*_.

The rock failure image under local load is transformed into a gray image, and cracks are identified by the image gray principle. The *D*, *K*_*f*_, and *R*_*f*_ of jointed rocks under different loading areas are calculated by Eqs (7), (9), and (10), respectively. The results are shown in Fig 20.

The curved surfaces in Fig 20 are fitted by the theoretical results. The loading area is the main factor affecting *D*, *K*_*f*_, and *R*_*f*_ of jointed rock cracks. The larger the loading area, the more complex the cracks, the more random the distribution of cracks, and the higher the penetration rate of cracks. The joint angle is another factor affecting *D*, *K*_*f*_, and *R*_*f*_ of rock cracks, though its degree of effect is small. Full area S is the only loading condition under which *D*, *K*_*f*_, and *R*_*f*_ of rock cracks increase significantly at the joint angle of 0° or 90°, indicating the complexity and distributional randomness of cracks, the high penetration rate, and the severe rock failure at either angle.

## Conclusions

Although jointed rock masses are common in nature, existing research on the mechanical behavior of jointed rocks has ignored the effect of loading area. This study has examined the dual effects of loading area and joint angle on the mechanical behavior and failure characteristics of a jointed rock using UDEC software. Below are the main conclusions:

1. Under the full-area load of S, *σ*_ci_, *σ*_cd_ and *σ*_cf_ of jointed rocks have little difference at the same absolute value of joint angles. Under local load, the strength dividing points of rocks with negative-angle joints are greater than those with positive-angle joints at the same absolute value of joint angles. *σ*_ci_, *σ*_cd_ and *σ*_cf_ of non-persistent jointed rocks bear positive relation to loading area, with *σ*_cd_ and *σ*_cf_ more affected than *σ*_ci_ by loading area.
2. The curves of relationship between joint angles and *U* at the peak, *U*_e_ at the peak and the amplitude of post-peak abrupt change of energy all present W-shape distributions. The difference of energy between rocks with different angle joints decreases with the shrinkage of the loading area. The effect of loading area on *U* is dominant.
3. Once the non-persistent jointed rock is damaged, tensile cracks outnumber shear cracks. In the post-peak failure stage, shear cracks predominate under the load areas of S, 0.75S, and 0.5S, whereas tensile cracks predominate under the 0.25S loading condition. As joint angle *α* approaches 0°, the macro failure type of the joint tip of rocks transforms from tensile-shear failure to tensile failure.
4. The larger the loading area, the more complex the cracks in the failure of jointed rocks, the more randomly the cracks are distributed, and the higher the penetration rate of cracks. However, joint angle has insignificant effects on fractal dimension *D*, crack entropy *K*_*f*_, and penetration rate *R*_*f*_ of rock cracks. Furthermore, full area S is the only loading condition under which *D*, *K*_*f*_, and *R*_*f*_ of rock cracks increase significantly at the joint angle of 0° or 90°.
